# Academic profile, beliefs, and self-efficacy in research of clinical nurses: implications for the Nursing Research Program in a Magnet Journey™ hospital

**DOI:** 10.1590/S1679-45082013000400018

**Published:** 2013

**Authors:** Eliseth Ribeiro Leão, Olga Guilhermina Farah, Elisa Aparecida Alves Reis, Claudia Garcia de Barros, Cristina Satoko Mizoi

**Affiliations:** 1 Hospital Israelita Albert Einstein, São Paulo, SP, Brazil

**Keywords:** Clinical nursing research, Nursing research, Nursing evaluation research

## Abstract

**Objective:**

To describe the academic profile, research experience, beliefs, and self-efficacy in research of clinical nurses in a Magnet Journey™ hospital.

**Methods:**

Quantitative descriptive designed to assess research experience of clinical nurses. The survey was divided into demographics characteristics; scientific/academic profile (Nursing degree; membership in academic research groups, involvement in papers, teaching activities, scientific conferences, and posters presented); beliefs related to nursing research (about skills, benefits to career, reputation of institution, patient care; job satisfaction level); and Research Self-Efficacy (conducting literature review; evaluating quality of studies; using theory; understanding evidence; and scientific writing: putting ideas on paper easily; recognize and adapt the text to the reader; write to the standards required by science; write with objectivity, logical sequence, coherence, simplicity, clarity, and precision; insert the references in the text correctly; write the references appropriately; use correct spelling and grammar; write texts in English).

**Results:**

Most clinical nurses had low research experience, yet had positive beliefs in and perception of well-developed research skills.

**Conclusion:**

Our findings should contribute to the preparation of research programs aimed at facilitating the engagement of clinical nurses in the development of scientific projects.

## RESUMO

**Objetivo:**

Descrever o perfil acadêmico, a experiência em pesquisa, as crenças e a autoeficácia em pesquisa dos enfermeiros clínicos de um hospital em Jornada Magnet^®^.

**Métodos:**

Estudo descritivo, tipo *survey*, para avaliar perfil acadêmico, experiência em pesquisa, crenças e habilidades de pesquisa dos enfermeiros clínicos. A análise foi dividida em características demográficas; perfil acadêmico/científico (titulação acadêmica, participação em grupos de pesquisa, envolvimento em publicações, atividades de ensino, conferências científicas e apresentação de posteres); crenças relacionadas à Pesquisa em Enfermagem (habilidades, benefícios para a carreira, reputação da instituição, para o cuidado do paciente, satisfação no trabalho); e autoeficácia em pesquisa (conduzir revisão de literatura, avaliar a qualidade dos estudos, usar teoria; compreender as evidências e escrita científica: facilidade para colocar as ideias no papel, reconhecer e adaptar o texto para o leitor, escrever obedecendo os padrões requeridos pela ciência, escrever com objetividade, clareza e precisão; inserir as referências apropriadamente, usar corretamente a ortografia e gramática; escrever textos em inglês).

**Resultados:**

A maioria dos enfermeiros clínicos tinha pouca experiência em pesquisa, todavia, demonstraram crenças positivas e percepção de habilidades de pesquisa bem desenvolvidas.

**Conclusão:**

Nossos achados devem contribuir para a elaboração de programas de pesquisa que objetivem facilitar o engajamento dos enfermeiros clínicos no desenvolvimento de projetos científicos.

## INTRODUCTION

Nursing care is characterized by technical and humanistic competence, as well as scientific knowledge in the field. High quality of care depends on all these dimensions.

One well-established way to promote these abilities is through the support and motivation of nursing research. Contact with scientifically structured information should enable nurses to be constantly updated in the field, evaluate their practices, and help them grow professionally by incorporating new ways of thinking and caring for their patients^(1)^.

A number of factors have contributed to the implementation of nursing research programs at private institutions, many of which related to the need to establish international quality standards in these centers. One such case of growing support for the development of nursing research is that derived from the requirements associated with the Magnet™ designation. The Magnet Recognition Program™ was developed by the American Nurses Credentialing Center (ANCC) to recognize healthcare organizations that have excellence in nursing services^(2)^. In Magnet™ designated hospitals or in Magnet Journey™ hospitals, research units are created to facilitate the development of research. But this is only a first step in the process. The support of a nurse-researcher (PhD-prepared nurse scientists) is always necessary^(3)^.

Building a culture of research outside the academic world is not an easy task, especially for professionals with full dedication to nursing care. Usually nursing research is still very restricted to universities and clinical nurses have many difficulties in developing research projects. First, how to implement research programs that promote the engagement of clinical nurses in nursing research? It is necessary to know the academic background of nurses, as well as their beliefs and self-efficacy in research to adopt the most appropriate strategies. Additionally, there is no consensus yet on the best model for training and research support, or on how to effectively engage clinical nurses in research. A comprehensive, multifaceted program is thus necessary to bridge this research-practice gap^(4)^. This is only possible through a detailed diagnosis of the background, experience, and beliefs of clinical nurses regarding research.

In this study we report the results of a detailed assessment of the academic profile, research experience, beliefs, and self-efficacy in research of clinical nurses employed by *Hospital Israelita Albert Einstein* (HIAE), at São Paulo, Brazil, which is the first organization in Latin America to request the Magnet™ designation. The understanding of the profile and idiosyncrasies of this nursing population enabled the preparation of a research program aimed to facilitate the engagement of clinical nurses in the development of scientific projects. Knowing the factors associated with the successful implementation of research practices in nonacademic centers, particularly those seeking the Magnet™ designation, can inspire other organizations in other contexts to initiate or consolidate this activity in bedside care.

## OBJECTIVE

To diagnose the academic profile, research experience, beliefs, and self-efficacy in research of clinical nurses in a Magnet Journey™ hospital.

## METHODS

A descriptive electronic survey was conducted using a convenience sample of 165 clinical nurses (20% of total clinical nurses as of January 2012) from HIAE, the first Latin American private hospital admitted into the Magnet Journey™ in October 2011. The invitation to participate and the questionnaires were sent by e-mail, participation was voluntary, and the data were processed in September 2012. All participants signed an Informed Consent Form for this study.

The survey was divided into four subsections: (1) Demographics (7 questions); (2) Scientific/Academic Profile (18 questions); (3) Beliefs Related to Nursing Research (8 questions; Likert Scale) as based on statements proposed by Apleton et al.^(5)^; and (4) Self-Efficacy in Research (22-item, 5-point response scale) as proposed by Sweson-Britt and Reineck^(6)^. The activities listed in the Nursing Research Self-Efficacy Scale were translated into Portuguese and added one item related to literature search in Latin America databases and one related to English writing (eight-item: putting ideas on paper easily; recognizing and adapting the text to the reader; writing with the standards required by science; writing with objectivity, logical sequence, coherence, simplicity, clarity, and precision; inserting the references into the text correctly; writing the references appropriately; using correct spelling and grammar; writing texts in English). This instrument does not grade scores and therefore does not require validation, since it only serves as a guide for the simple quantification of skills.

Categorical variables were described as absolute frequencies and percentages. Qualitative variables were expressed as median ranges. Comparisons between measures of beliefs and skills in different categories of profile variables were performed using the Mann-Whitney and Kruskal-Wallis tests.

For scores of beliefs and skills, the responses of items were added, and the score values were as follows: minimum score = 1 x number of items = maximum agreement or belief; maximum score = 5 x number of items = minimum agreement or belief. Thus, the minimum and maximum standardized scores were 0 and 10 (regardless of the number of items added), representing, respectively, the minimal and maximal levels of agreement or belief. Statistical tests were performed with Statistical Package for the Social Sciences (SPSS), version 17.0. Significance was considered at p≤0.05.

The Research Ethics Committee approved the study under number 36,268.

## RESULTS

### Academic profile of participants

Of the 165 survey participants, the majority (87.3%) were staff nurses, followed by nursing managers (6.1%), nurse administrators (5.5%), and research nurses (1.2%). Although all participants had a nursing degree, most (72.7%) had obtained their degree 5 years or more prior to the interview. Most participants (85.5%) also had a specialization degree. A total of 12.7% had a master's degree and 3% had a PhD.

The analysis of research experience revealed that only 13.3% participated in academic research groups. Yet, most participants reported reasonable and good experience (40% and 17.6%, respectively), with only 8.5% and 33.9% reporting no research experience or poor research experience. Similarly, the analysis shows that only 17.6% presented research results in scientific conferences and 9.7% had published a manuscript in the 2 years prior to the interview. Most (77.5%) respondents expected to participate in research activities. Participation in teaching activities was reported by about a third (34.5%) of respondents.

### Beliefs of clinical nurses and their impact on nursing research

Table 1 shows that most clinical nurses had positive beliefs about the benefits of nursing research, predominantly about the research impact on corporate image, in the development of team work, and in patient care.

**Table 1:** Beliefs regarding the impact of nursing research in quality of care and in working life

Beliefs	I totally believe n (%)	I believe n (%)	I neither believe or disbelieve n (%)	I do not believe n (%)	I do not totally believe n (%)	Total n (%)
Ensure standards of patient care are improved	84 (50.9)	75 (45.5)	6 (3.6)	0 (0)	0 (0)	165 (100)
Benefit staff in terms of developing their skills	83 (50.3)	78 (47.3)	4 (2.4)	0 (0)	0 (0)	165 (100)
Help improve the way the staff work together	76 (46.1)	83 (50.3)	6 (3.6)	0 (0)	0 (0)	165 (100)
Raise the profile and reputation of the unit	89 (53.9)	68 (41.2)	6 (3.6)	2 (1.2)	0 (0)	165 (100)
Raise the profile and reputation of the centre	97 (58.8)	65 (39.4)	3 (1.8)	0 (0)	0 (0)	165 (100)
Help me advance in my professional development/career	103 (62.4)	56 (33.9)	5 (3.0)	1 (0.6)	0 (0)	165 (100)
Promote patient and care involvement in the way the ward is organized and run	63 (38.2)	77 (46.7)	17 (10.3)	7 (4.2)	1 (0.6)	165 (100)
Make my job more satisfying	79 (47.9)	68 (41.2)	12 (7.3)	6 (3.6)	0 (0)	165 (100)

### Self-efficacy in nursing research

**Figure 1:**
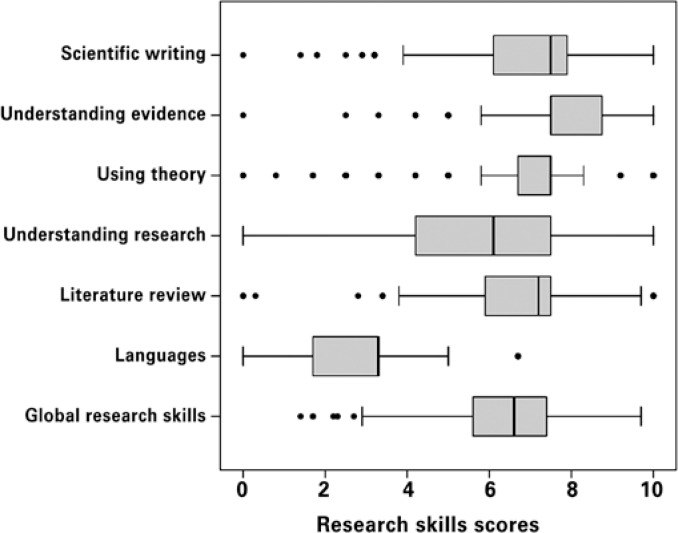
Research skill scores reported by 165 clinical nurses of *Hospital Israelita Albert Einstein* using the Nursing Research Self-Efficacy Scale (adapted from NURSES). Sao Paulo, Brazil. September, 2012

Figure 1 shows higher scores on skills related to using theoretical knowledge, understanding evidence, and the ability to perform literature reviews. The ability to critically analyze scientific articles in other languages was not much evaluated.

The analysis of the global research skills of this population indicated that these skills depended on the degree of complexity of each research activity. Concordance rates (answers ‘agree’ and ‘totally agree’) as related to the ability to review the literature ranged from 51.2% to 87.3% (mean 68.7%). The scores attributed to critical analysis skills ranged from 38.1 to 60.6% (mean 49.5%), and those attributed to theoretical and evidence-based practice ranged from 75.1% to 89.7% (mean 82.2%). Scientific writing abilities had scores that ranged from 67.3 to 84.2% (mean 73.6%). Most respondents believed they were able to read and understand a scientific article in English or in Spanish (70.9 and 69.1%, respectively). However, few nurses were capable of writing an article in these languages (23.6 and 7.9%, respectively). Only 2.4% were able to read and understand French, and the ability to write in that language was not observed.

Poorer writing abilities were significantly associated with a longer time since graduation (p=0.008). As expected, better developed writing skills were associated with a graduate degree (p=0.022), and having a PhD degree was associated with a higher general ability (p=0.044) and a better developed ability for research (p=0.018). Teaching also favored positively the general research skills of these nurses (p=0.011), as well as their ability to search for information and use theoretical and evidence-based practice skills (p=0.004, 0.031, and 0.002, respectively). Their academic institution of origin (public or private), participation in research training courses, and having their research results in publications or events presented by nurses were not statistically significant in relation to research skills.

Nurses with a master's degree had more positive beliefs about the benefits of research and better developed research skills (Table 2). Participation in research groups was the most influential factor in this regard (Table 3). Similarly, having presented research results in scientific events positively affected the beliefs of nurses regarding research (p=0.001), whether they were the principal investigator (p=0.002) or co-author (p=0.032). A total of 77.5% of respondents expected to carry on research in the future, and 37.5% demonstrated interest in innovation processes.

**Table 2:** Association between beliefs/skills and Master's Degree

Beliefs and skills	Master's degree	No Master's degree	p value
	Median	Median	
Beliefs (0–10)	10	8.4	<0.001
General skills (0–10)	7.4	6.5	0.011
Languages (0–10)	3.3	3.3	0.001
Conducting literature review (0–10)	7.5	6.9	0.018
Understanding research literature and evaluating the quality of studies (0–10)	6.7	5.8	0.121
Using theory (0–10)	7.5	7.5	0.033
Understanding evidence (0–10)	7.5	7.5	0.051

**Table 3:** Association between beliefs/skills and participation of clinical nurses in academic research group

	Yes	No	p value
	Median	Median	
Beliefs (0–10)	10	8.4	<0.001
General skills (0–10)	7.1	6.6	0.049
Languages (0–10)	3.3	3.3	0.062
Conducting literature review (0–10)	7.5	6.9	0.017
Understanding research literature and evaluating the quality of studies (0–10)	6.3	5.8	0.679
Using theory (0–10)	8.3	7.5	0.006
Understanding evidence (0–10)	10	7.5	<0.001
Scientific writing (0–10)	7.1	7.5	0.488

## DISCUSSION

In this study a detailed profile of the research experience, beliefs, and self-efficacy in research of clinical nurses was established in the first Magnet Journey™ hospital in South America.

Varying degrees of research experience were reported by the 165 respondents. By and large, clinical nurses were overwhelmingly positive in their beliefs, as well as in their perception of their own research skills, which was more positive than previously reported^(6,7)^. It was observed that the nurses who attended specialization courses had a higher perception of the difficulties associated with scientific writing. Perhaps they had a more critical view of their own limitations through having been exposed to this experience. On the other hand, some barriers, such as insufficient time to conduct research, may have hindered the ability of these professionals to perform and publish research^(8,9)^. Also, because participation in publications reinforced such positive beliefs, but not the perception of their own research skills, these results indicate that clinical nurses may have not been the principal investigators of the studies published.

Another aspect that may have contributed towards low scientific production is the difficulty in critically analyzing texts and scientific articles written in other languages. Indeed, the ability of critical analysis of scientific texts written in English was not much evaluated by our respondents. English is the international language of science^(10)^, but for authors whose mother tongue is not English, writing scientific manuscripts represents a great challenge.

It has been previously suggested that clinical nurses are highly motivated to participate in research, but lack confidence in the initiation and implementation of research projects^(11)^. With few exceptions, nursing research has not been a typical part of clinical nursing activities. It is rare to have personnel with the skills and educational background necessary to facilitate research^(7)^. Many clinical nurses feel intimidated by research. Prior research is in line with this study, having reported that nursing staff have a “fear of the unknown”, “lack the education or confidence to conduct research”, or “the belief that they do not have skills to accomplish research (which) prevents them from pursuing research projects”^(12)^.

In this study, most nurses perceived the following skills to be well developed: the ability to search for information effectively, theoretical skills, evidence-based practice, writing and reading abilities, and comprehension of English and Spanish. The completion of a master's or doctoral degree, as well as participation in research groups and teaching, also had a positive influence on the nurses' perception about their skills. Self-perception of clinical nurses about their research skills was in fact higher than previously reported^(6,7)^. Nevertheless, our results indicate that these skills seemed to favor evidence-based practice more than nursing research. The low scientific production reported here reinforces this idea, in agreement with the notion that only isolated nurses carry on research, and publication is infrequent^(13)^.

Our findings also indicate that the main impact of nursing research perceived by the respondents is associated with improvement of the organizational image. This value is strongly recognized by employees of the institution. This can be explained by the fact that our hospital is recognized for excellence in quality care, and is a top institution in Brazil. In 2012, the HIAE was recognized by the SciVal Brazil (Elsevier) Award, which enshrines Brazilian research institutions of outstanding excellence in scientific production. HIAE received the award in the category “Quotes by document.” Our findings are in contrast with prior reports^(5)^ indicating that the most positive responses of clinical nurses about their views on the impact of research are linked to standards of patient care and the development of specific skills.

Conducting and applying researches have not been a priority for the average staff nurse, despite the widely accepted belief that nursing research is relevant. While most clinical nurses demand evidenced-based practice, they are apparently reluctant and apathetic as to performing nursing research^(13)^. Indeed, nurses in both academic and nonacademic healthcare settings have reported low to moderate interest in research, suggesting that this is a universal nursing issue^(4)^. Our results support this notion, as evidenced by the very fact that the spontaneous level of adherence of clinical nurses to respond to the questionnaires was low. For most nurses, the research theme seemed too distant and aroused little interest. The recent hiring of a nurse researcher and unfamiliarity with a new department (Nursing and Multidisciplinary Research Office) that previously did not exist in the institution may also have contributed to that. The observation that nursing research is not a priority for clinical nurses is unaccountable, as hospital-based nurses are well positioned to identify clinical practice problems encountered on a daily basis at the bedside, and could help generate evidence for their practice^(12)^. Here, little participation in research groups was observed, hence little research experience as well. This is consistent with prior analyses showing that nurses want to partner with nursing schools to conduct research^(14)^. Nevertheless there are several challenges, such as difficulty in negotiating research proposals of common interest to university faculty and staff nurses. Some nurses would prefer to lead their own research rather than partner with nursing schools^(15)^. In particular, clinical nurses report wanting to perform studies that translate into real-life benefits.

Overall, our diagnosis of the academic and scientific profile of clinical nurses registered at the HIAE indicated a high heterogeneity of profiles, as well as some idiosyncrasies and beliefs among this population that should be considered in the implementation of a program tailored towards the development of nursing research practices. One limitation of the present study, however, is the fact that the respondents may not represent the profile of the clinical nurses of the organization. The participants who chose to complete the survey might have an increased awareness of the relevance and value of nursing research for practice.

Several challenges remain for the development of the best institutional structures supporting and integrating research in the clinical setting while making research findings relevant to practicing clinicians. The most common model structures include the presence of a hospital-based nursing council with guidance from academic researchers; a contracted academic nurse researcher; a part-time nurse researcher; and a fulltime hospital-based nurse researcher^(16)^. The last model is adopted in our institution. The Nursing and Multidisciplinary Research Office was established to promote and support research practices and training among clinical nurses and the hospital's multidisciplinary team. At HIAE, the nurse researcher plays many roles: independent researcher, teacher/coach, consultant, and mentor, as similarly reported in other institutions worldwide^(17)^.

The diagnosis of the academic profile of nurses identified here indicates that different types of training programs are necessary. Therefore, we propose the following training program: basic training levels involving courses on scientific methodology (8 hours), bibliographic search (regular library programs), and scientific writing (16 hours). For intermediate and advanced levels of experience, we recommend the critical analysis of articles and scientific writing (40 hours). For continuing education we propose the implementation of monthly meetings. Additionally, our program gives a research award for clinical nursing scientific contributions and has implemented strategies for their increased visibility. These scientific contributions are assessed through quantifiable indicators (articles published, citations, innovation proposals, patents, and collaborative projects).

The improvement of the research skills of clinical nurses is debated worldwide. Although the present study was carried out in a private hospital in Brazil, our findings may be useful to other organizations with similar profiles in different regions and countries. Similarly, our proposed data-based nursing research program may also contribute to future discussions on the implementation of nursing research in nonacademic institutions, particularly those seeking to obtain the Magnet™ designation. Understanding the research experience, skills, and beliefs of the clinical nursing staff of an Institution can help the implementation of research programs, as well as the selection of the best training strategies. Such an analysis is always needed to reduce the risk of importing inappropriate actions from other studies, which may not correspond to the institutional reality. There is no ideal model described in the literature to improve the involvement of clinical nurses in nursing research. However, it is important to reflect on the ways that lead to the effective development of clinical nurses in nursing research, and consider the profile of nurses carrying on bedside research. Not all clinical nurses have the desire and aptitude for the task. Additionally, no study is available to date that enables identifying the optimal ratio of research nurses to total nursing personnel. In the future, there may be recognition of the need to hire additional nurse researchers, not only for support or mentoring, but also to develop high-quality research. This will be a real transformation in how scientific knowledge is produced by nurses in hospitals, and will enable the vocation of every nurse to be properly evaluated and followed.

Our findings should contribute to the elaboration of a research program proposal tailored to the level of academic experience of this population.

## CONCLUSIONS

In this study a detailed profile of the research experience, beliefs, and self-efficacy in research of clinical nurses was established in the first Magnet Journey™ hospital in South America. Varying degrees of research experience were reported by clinical nurses. Positive beliefs and perceptions about research skills were observed. However, positive perception was not translated into effective scientific production.
